# Outcomes in Child Health: Exploring the Use of Social Media to Engage Parents in Patient-Centered Outcomes Research

**DOI:** 10.2196/jmir.6655

**Published:** 2017-03-16

**Authors:** Michele P Dyson, Kassi Shave, Ricardo M Fernandes, Shannon D Scott, Lisa Hartling

**Affiliations:** ^1^ Alberta Research Centre for Health Evidence Department of Pediatrics University of Alberta Edmonton, AB Canada; ^2^ Clinical Pharmacology Unit Instituto de Medicina Molecular University of Lisbon Lisbon Portugal; ^3^ Department of Pediatrics Santa Maria Hospital Lisbon Portugal; ^4^ Faculty of Nursing University of Alberta Edmonton, AB Canada

**Keywords:** social media, child health, patient outcome assessment, patient engagement

## Abstract

**Background:**

With the rapid growth of technology and its improved accessibility globally, social media is gaining an increasingly important role in health care. Patients are frequently engaging with social media to access information, share content, and interact with others in online health communities. However, the use of social media as a stakeholder engagement strategy has been minimally explored, and effective methods for involving participants in research on the identification of patient-centered outcomes remain unknown.

**Objective:**

The aim of this study was to evaluate the process of using social media to engage parents in identifying patient-centered outcomes, using acute respiratory infections in children as an example to gauge feasibility.

**Methods:**

We conducted a process evaluation of a two-phase Web-based strategy to engage parents in research on patient-centered outcomes. In the first phase, we developed a website and study-specific Facebook and Twitter accounts to recruit parents to complete a Web-based survey identifying patient-centered outcomes. In the second phase, we used Facebook to host discussion with parents based on the survey results. The reach of social media as an engagement strategy and the characteristics of the population recruited were assessed.

**Results:**

During the first phase, there were 5027 visits to the survey site, 110 participants completed the survey, 553 unique users visited the study website (675 visits), the Facebook page received 104 likes, and the Twitter account gained 52 followers over the 14-week study period. Most survey respondents identified Facebook (51.8%, 57/110) or a friend (45.5%, 50/110) as their source of referral. It was found that 70.0% (77/110) of respondents resided in Canada, in urban centers (92.7%, 102/110), and 88.2% (97/110) had a college or university degree or higher. The median year of birth was 1978 and 90.0% (99/110) were female. Most survey responses (88.2%, 97/110) were completed during the first month of the study. In the second phase, 4 parents participated in the discussion, our Facebook page gained 43 followers, and our posts reached 5866 users. Mirroring the first phase, most followers were female (79%, 34/43), between 35 and 44 years (49%, 21/43), and resided in Canada (98%, 42/43). User engagement was variable, with no clear patterns emerging in timing, topic, or type of post.

**Conclusions:**

Most participants were highly educated, urban dwelling, and female, limiting diversity and representativeness. The source of referral for most survey respondents was through Facebook or a friend, suggesting the importance of personal connections in the dissemination and uptake of information. User engagement with the Facebook-based discussion threads was inconsistent and difficult to initiate. Although there is substantial interest in the use of social media as a component of an effective patient engagement strategy, challenges regarding participant engagement, representativeness, obtaining buy-in, and resources required must be carefully considered.

## Introduction

### Health Care

With major technological advances occurring in the past decade, the way patients seek out and engage in their health care is shifting. Nearly 3.5 billion people worldwide have access to a computer or mobile device at home, making a wealth of largely unvetted information widely available [1]. In health care, the Internet is gaining an increasingly important role, and 72% of American Internet users reported going online to seek out health information in 2012 [2]. Patients are using social networking websites to access health information from a wide variety of sources, post content about their personal health, and interact with others in online health communities [3]. This environment of open creation and exchange of user-generated content represents a significant evolution in the way information can be communicated and developed, and presents an opportunity for conducting research with an existing audience of engaged patients [4].

Along with its widespread use among health professionals and patients, the body of published literature examining the role of social media in health care is growing. Recent systematic review data have underscored the potential benefits, ethical issues, and unintended consequences of using social media in the health care environment [5-7]. For example, although online communities may represent an important source of social and emotional support for patients, participation in such communities may also result in diminished subjective well-being (eg, increased anxiety), and invoke concerns for privacy. Social media use may also impact, both positively and negatively, the relationships that patients have with their health care providers [8]. Despite the recent proliferation of literature with respect to the utility, benefits, and harms of social media use in health care, far less information exists with respect to utilizing social media to recruit and engage patients in health research.

The active engagement of patients in the research process is important, as it can improve on the credibility of the results and their relevance to the end users, thus reducing research waste [9]. Moreover, a number of studies have found that engaging patients in research improves rates of study enrollment and decreases losses to follow-up [9]. Within the context of patient engagement, patient-centered outcomes research seeks to elicit patients’ unique perspectives on outcomes that are important to themselves and their families [10]. Patient-centered outcomes research, by definition, relies on engagement and input from patients themselves; however, participating in research may not be attractive for myriad reasons, including perceived and actual participant burden. Among the patient population, garnering interest in and excitement about research that will affect their own lives is crucial; however, knowledge of the optimal methods of engagement remains limited.

### Social Media

Social media represents an innovative and potentially efficient method to attract attention, collect information, and generate dialogue from a widespread audience including patients [6,11]. Next to YouTube, Facebook and Twitter are the most accessed social media apps globally [12], thus representing opportune platforms to engage and recruit difficult-to-reach populations traditionally underrepresented in research [13-15]. Once engaged, effectively using social media to stimulate user-generated content can help to broaden the reach of a study through expanding personal and professional networks [16]. As participants engage in the study, people in their social networks are notified and may feel enticed to participate. Interactivity (eg, tweets, retweets, likes, comments, shares) may also build interest in and credibility of the study platform and subsequently increase followers.

In 2013 and 2016, we used social media to conduct two phases of recruitment for a mixed methods study (the OUTcomes in Child Health, OUTCH study) to understand parent perceptions of patient-centered outcomes for pediatric acute respiratory infections (ARIs), which represent a clinical area that is common among children and has a significant impact on the health care system, and patient and family well-being. Furthermore, ARIs are associated with significant morbidity and are one of the leading causes of illness and hospitalization in children [17]. Despite this burden of disease, there is a scarcity of research on families’ perspectives of having a child with an ARI. Parents are a growing group of social media users [18,19], and given the breadth of our scope (ARIs; parents of children 0-17 years), represent a suitable audience to recruit through social media platforms.

In this paper, we evaluate the effectiveness of using social media as a strategy to recruit and engage with parents in determining patient-centered outcomes in a mixed methods study. Our specific objectives were to (1) systematically evaluate the process of using social media as a stakeholder engagement strategy and (2) examine the breadth and representativeness of the population engaged.

## Methods

### Study Design

Our mixed methods study, utilizing the sequential explanatory design [20], was conducted in two phases: an initial quantitative survey was conducted in which parents were asked to rate the importance of outcomes relevant to pediatric ARIs, followed by a qualitative follow-up phase in which we sought further elaboration from parents on their rationale for their rating of outcomes for pediatric ARI. Ethics approval was granted for both phases by the University of Alberta Research Ethics Board; consent was implied through overt action on the part of participants. The methods for our social media engagement strategy for each phase of the study are described below.

### Phase I: Utilizing Social Media to Recruit and Engage Parents in a Quantitative Survey

**Figure 1:**
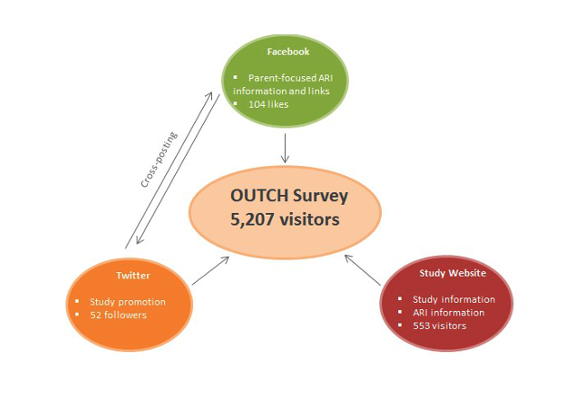
Flow diagram outlining the online components of phase I of the OUTcomes in Child Health (OUTCH) study.

Our initial engagement strategy consisted of a suite of Web-based tools that we used as a hub to recruit potential participants and direct them to an open and voluntary Web-based survey on patient-important outcomes in pediatric ARI. We developed a Web presence with three components developed to promote the study and drive traffic to the survey: a study website [21] (see Multimedia Appendix 1) to host information about the study and conditions of interest; a Facebook page (OUTCH) [22] (see Multimedia Appendix 2) to post information and links related to pediatric ARI that would be of interest to parents; and a Twitter account (@OUTCH_Study) [23] (see Multimedia Appendix 3) to expand our network and cross-post content from our Facebook page. Although each of these platforms was used to present information in different formats, the primary goal of each was to engage parents with the aim of recruiting them as study participants, and direct them to the unique survey website (Figure 1).

Creating our own social media presence was important to establish a recognizable brand and to ensure credibility. We had direct control over the appearance and content on our website, which was necessary for ethical considerations, and employed consistent messaging throughout our channels. Although existing social media communities provide the advantage of an established group of followers, we sought to understand the opportunities and challenges associated with building a community of patients and families who share a common interest in informing patient-relevant research. Also, by relying on other channels to advertise one’s study, control over the messaging is lost, increasing the potential for miscommunication, and increasing the complexity of tracking activity specific to our engagement strategy. Because social media, by its very nature, is interactive, we still expected to garner sufficient interest by engaging known, credible sources in our recruitment strategy. We also felt that having our own channels would facilitate continued active patient engagement moving forward.

We recruited parents through a snowball sampling technique [24], initially focusing on recruitment targets with the potential for a high yield of participants and then expanding through referrals and diffusion via social media. We targeted key local, national, and international English-speaking organizations with an interest in ARI and patient engagement, including TRanslating Emergency Knowledge for Kids (TREKK; research network with a Parent Advisory Group), the Cochrane Consumer Network (CCNet; organization that involves health care consumers in systematic reviews), the Alberta Centre for Child, Family & Community Research (ACCFCR; center that links government, academia, and the community), the Stollery Family Centered Care Network (network of families associated with a local children’s hospital), and Mommy Connections (online parenting community) and asked them to promote the study to their audiences. We also built our audience by strategically seeking out followers (Twitter) or likes (Facebook) from individuals and organizations that emphasized parenting, health care, or local interest. Our social media platforms were intended to stimulate parent engagement and we posted daily content through both Facebook and Twitter. Facebook posts included information and links related to pediatric ARI that could be useful to parents. Posts on Twitter advertised the study and were linked to the Facebook page so that content was highlighted through both accounts. As an incentive, we included a raffle in which respondents could enter their names into a draw for an iPad mini, Kobo Touch, or Nike+ FuelBand.

We partnered with an online marketing company for the last 4 weeks of the 14-week survey period to evaluate the impact of paid advertising strategies on our recruitment efforts. We implemented a Facebook advertising campaign with standard ads (found along the right-hand side of the page) and page post ads (appear in the main News Feed), using an A/B testing format (head-to-head comparisons) to determine the highest performing ads. These evaluations were conducted over the course of the advertising campaign and the results were used to modify and target existing ads to maximize effectiveness.

### Phase II: Utilizing Social Media to Recruit and Engage Parents in Qualitative Follow-Up

In the second phase, our goal was to engage parents in discussion on our Facebook page, and our recruitment efforts focused almost exclusively on promotion through Facebook, which was chosen as our primary interface for interaction with parents based on our experiences and relative success with the platform over Twitter in phase I. We built in a 2-week promotional period in which all posts were focused on publicizing the upcoming study. During this phase, some of our affiliated research partners used Twitter to promote the study; however, all traffic was directed to our Facebook page. Over an 8-week study period, we used Facebook to host an online discussion forum in which we asked parents about the outcomes that matter to them when their child is ill with an ARI. Each week covered a new topic, with daily posts throughout the week, including discussion prompts and parent-friendly content related to respiratory infections in children. The discussion was moderated, with every comment receiving a follow-up response from the study team. To develop our network, we contacted several groups and organizations relevant to parents and child health in advance in an effort to obtain buy-in and encourage promotion of our study. These included online parenting communities (n=16), children’s hospitals and associated foundations (n=14), and patient groups (n=3), both at the local and national levels. We also encouraged existing research networks and collaborators to help advertise our study (TREKK; Canadian Association of Paediatric Health Centres [CAPHC]; Knowledge Translation (KT) Canada; Alberta Research Centre for Health Evidence [ARCHE]). One of the parenting communities (Modern Mama) agreed to regularly share our posts for the duration of the study period for a nominal fee; any other engagement was informal and ad hoc.

### Data Analysis

We measured user engagement with each of the online components through a series of metrics detailing reach and engagement from the study website (Google Analytics), survey website (Nooro), Facebook page (Facebook Insights), and Twitter account (followers). We also collected demographic information from our survey and available Facebook and Twitter data to examine the composition of our sample. We used descriptive statistics to analyze the data.

## Results

The findings that describe the process evaluation of our social media engagement strategy are presented below, according to study phase. Data collected on patient-centered outcomes from the survey and qualitative follow-up are beyond the scope of this evaluation, and will be reported elsewhere.

### Phase I: Utilizing Social Media to Recruit and Engage Parents in a Quantitative Survey

Our survey was live for 14 weeks between December 2013 and March 2014, and we received 110 responses. The study website received 675 visits from 553 unique visitors; 5207 users visited the survey website; the Facebook account received 104 “likes;” and the Twitter account gained 52 followers. The most common sources of referral to the survey were Facebook (51.8%, 57/110), a friend (45.5%, 50/110), and Twitter (10.0%, 11/110).

The demographics of our sample are provided in Table 1. It was found that 90.0% (99/110) of respondents were female, the median year of birth was 1978 (range 1946-1995; age 38), and 88.2% (97/110) had a college or university degree or higher. Additionally, 70.0% (77/110) of respondents resided in Canada, and 92.7% (102/110) were based in urban centers.

**Table 1:** Characteristics of survey respondents in phase I (n=110).

Demographics		Response
**Gender, n (%)**		
	Female	99 (99.0)
	Male	11 (10.0)
**Year of birth, median (range)**		1978 (1946-1995)
**Highest level of schooling completed, n (%)**		
	Some high school	1 (0.9)
	High school graduate	5 (4.6)
	Some college or university	7 (6.4)
	College or university graduate	50 (45.5)
	Postgraduate education degree	47 (42.7)
**Marital status, n (%)**		
	Never married (single)	5 (4.6)
	Married or common-law	98 (89.1)
	Separated, divorced, or widowed	7 (6.4)
**Annual household income in US $, n (%)**		
	<30,000	5 (4.6)
	30,000-49,999	7 (6.4)
	50,000-69,999	15 (13.6)
	70,000-89,999	14 (12.7)
	>90,000	69 (62.7)
**Country of residence, n (%)**		
	Australia	2 (1.8)
	Canada	77 (70.0)
	England	8 (7.3)
	India	2 (1.8)
	United States	19 (17.3)
**Type of community, n (%)**		
	Urban (≥10,000 population)	102 (92.7)
	Rural (<10,000 population)	7 (6.4)
	Missing	1 (0.9)
**Relationship to the children in the family, n (%)**		
	Parent	106 (96.4)
	Step-parent	0 (0.0)
	Grandparent	4 (3.6)
	Other	2 (1.8)
**Number of children living in the home, median (range)**		2 (0-4)
**What illness or illnesses that affect breathing has your child or children ever had? n (%)**		
	Bronchiolitis	29 (26.4)
	Croup	49 (44.6)
	Strep throat or tonsillitis	36 (32.7)
	Sinusitis	32 (29.1)
	Wheezing	48 (43.6)
	Influenza	42 (38.2)
	Pneumonia	24 (21.8)
	Asthma	29 (26.4)
	Other	23 (20.9)
	Can’t remember	5 (4.6)
**How did you hear about this survey? n (%)**		
	Facebook	57 (51.8)
	Twitter	11 (10.0)
	Internet search	1 (0.9)
	Consumer group	4 (3.6)
	Friend	50 (45.5)
	Health care provider	3 (2.7)
	Research network	1 (0.9)
	Other	2 (1.8)

#### Study Website

On the study website, the home page received 88.2% (789/895) of all page views, with few users accessing the pages describing the study (6.6%, 59/895), the illnesses of interest (3.7%, 33/895), or study contact information (1.6%, 14/895). Engagement with the study website was typically brief, with 92.0% (621/675) of sessions resulting in drop-offs from the landing page, and 88.3% (596/675) of sessions lasting between 0 and 10 s. Most traffic (52.9%, 357/675) originated from the paid advertising campaign (described below), followed by 34.4% (232/675) from direct traffic (entering the specific Web address), and 11.1% (75/675) from social networks. Of the 75 visits that were derived from social media, 99% (74/75) were from Facebook and 1% (1/75) were from Twitter. Users accessed the website via a desktop computer in 44.6% (301/675) of cases, a mobile device in 37.6% (254/675), and a tablet in 17.8% (120/675). Of traffic originating from Facebook, 57% (42/74) of sessions were linked from the mobile site, and 43% (32/74) were from the main site.

#### Survey Website

**Figure 2:**
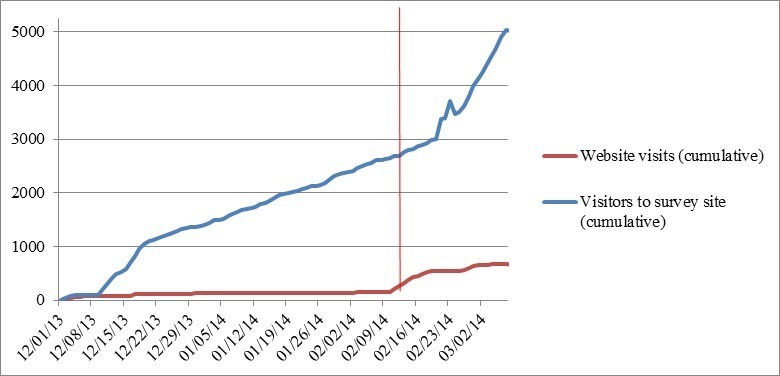
Web traffic to the study and survey websites (phase I). The red vertical line indicates the initiation of the advertising strategy.

Of 5027 visits to the survey website, 205 users opened the survey (4.08%, 205/5027). It was found that 37.1% (76/205) of surveys were blank, 9.3% (19/205) were partially completed, and 53.7% (110/205) were fully completed. On average, the 37-question survey took users 11.3 min (2 to 308.8 min) to complete. Of the surveys that were partially complete, all respondents completed the first page, and 16% (3/19) completed everything except the last section on demographics (18 questions). Data were not available on the origins of this traffic; however, traffic over time is depicted in Figure 2.

#### Facebook

Over the course of the study period, the study Facebook page accumulated 93 lifetime total likes (104 total likes, minus 11 “unlikes” during the study period). The mean number of page views from unique logged-in users was 2 per day (median 1; interquartile range [IQR] 0-2; range 0-12). The daily total reach, encompassing the number of unique users who have seen any content associated with our page (eg, posts, likes, and comments), ranged from 0 to 78,869 people (mean 9111; median 20; IQR 9 to 3711), with substantial variation between the pre- and postadvertising phases (described below). Although we were unable to capture much information on the individuals who liked our page due to the use of privacy settings and the transient nature of followers (ie, can rapidly cycle between “liking” and “unliking” the page), we were able to obtain geographic locations for a sample of 63 users. Of these, 51% (32/63) were located in the United States, 46% (29/63) in Canada, and 3% (2/63) in Portugal; 87% (55/63) were in urban centers (≥10,000 population). In total, 10 followers out of all users (11%, 10/93) were directly linked to the study team (study investigators, center staff, and advertisers).

#### Twitter

We had 52 followers on Twitter. Over the 96-day study period, our tweets (n=168) received approximately 7200 impressions, indicating the number of times users saw a tweet on Twitter. Our tweets received 32 link clicks and 61 retweets. As with the Facebook profiles described above, we were limited in the amount of information we could extract on Twitter followers, but of a sample of 58 ever-followers, including 17 who unfollowed the account, users’ profile descriptions primarily identified as a health care professional or researcher (n=22), an academic or professional organization (n=14), or a source of parenting or wellness information (n=7). Other categories included individual parents (n=5) and foundations or advocacy groups (n=2).

#### Impact of Advertising

**Figure 3:**
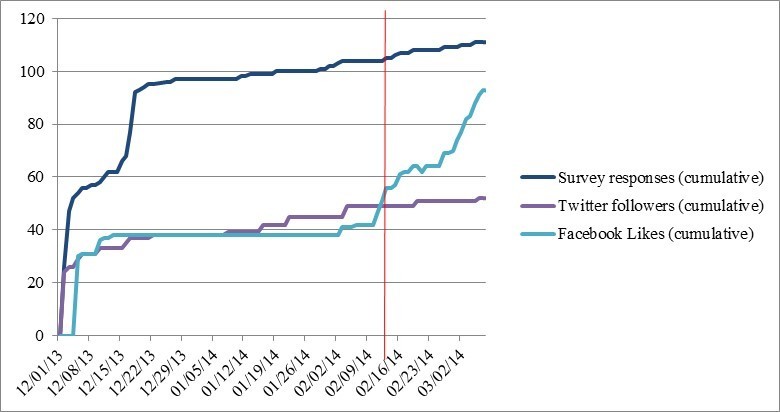
Survey responses and social media engagement over time (phase I). The red vertical line indicates the initiation of the advertising strategy.

Web traffic is depicted in Figures 2 and 3. In the 71 days before initiation of the paid advertising strategy, traffic to the study website was consistently low, with a mean of 2.3 visits per day (median 0; IQR 0-2; range 0-36). Our primary intention was to drive users to the survey website, and this was reflected with higher traffic, with a mean of 37.4 visits per day (median 30; IQR 18-46.5; range 0-133). Survey responses were highest in the first two days of the study period (42.7%; 47/110), with a mean response rate during the preadvertising phase of 1.5 per day. By the end of our initial study phase (preadvertising), 93.6% (103/110) of survey responses had been collected. Facebook likes and Twitter followers followed a similar pattern, with the sharpest rise at the beginning of the study, and activity leveling off after the first few days.

Our advertising strategy covered a 25-day period, in which 7 (6.4%; 7/110) respondents completed the questionnaire. The mean number of daily visits to the study website was 20.6 (median 10; IQR 3-5; range 0-61), and to the survey website was 94.8 (median 56; IQR 26-127; range 13-376). However, this encompasses a shift in emphasis. The first 10 days of advertisements were mistakenly driving traffic to the study website; after this period, clicks on the advertisements led directly to the survey website (Figure 2). Facebook likes increased during this phase (61 new likes), although engagement with the Twitter account remained static (3 new followers; Figure 3).

The total cost for our 4-week advertising campaign was CAD $1330 and reached an audience of an estimated 684,887 unique people. The advertisements received 1862 clicks that directed users to the study website or the survey. Overall, promoted posts were more effective than standard advertisements; and of the three highest performing advertisements (based on numbers of clicks and impressions), two were promoted posts and one was a standard advertisement. The top advertisement included content promoting our raffle.

### Phase II: Utilizing Social Media to Recruit and Engage Parents in Qualitative Follow-Up

We conducted our qualitative phase over a 2-week promotional and 8-week study period between January and March 2016. During this time, we published 52 posts, gained 43 Facebook followers (combined total for phases I and II: n=134), and attracted 4 participants to contribute to the discussion forum. Demographic data were collected from Facebook where possible; however, available data were limited by individuals’ use of privacy settings (Table 2). Closely following the observed patterns from the survey phase, 79% (34/43) of Facebook followers were female, 49% (21/43) were 35-44 years of age, 98% (42/43) resided in Canada, and 100% (43/43) were based in urban centers.

**Table 2:** Characteristics of Facebook page followers gained in phase II (n=43).

Demographics		Response n (%)
**Gender**		
	Female	34 (79)
	Male	6 (14)
	Undisclosed	3 (7)
**Age**		
	18-24	2 (5)
	25-34	14 (33)
	35-44	21 (49)
	45-54	5 (12)
	55-64	1 (2)
	65+	0 (0)
**Country of residence**		
	Canada	42 (98)
	Egypt	1 (2)
**Type of community**		
	Urban (≥10,000 population)	22 (51)
	Rural (<10,000 population)	0 (0)
	Undisclosed geographic location	21 (49)

#### Facebook Post Activity

**Figure 4:**
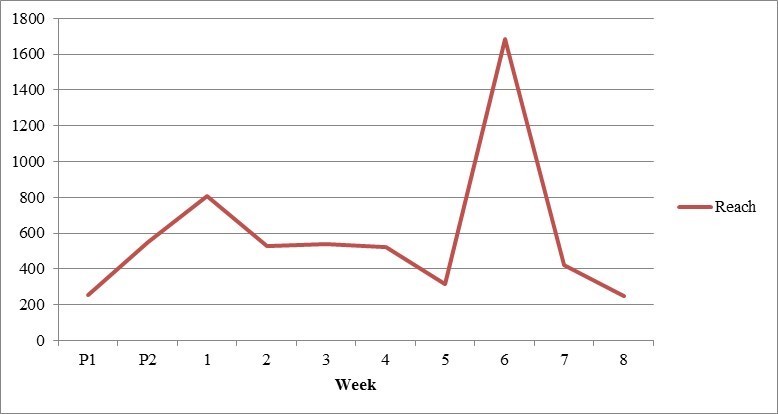
Weekly reach of Facebook posts (phase II).

**Figure 5:**
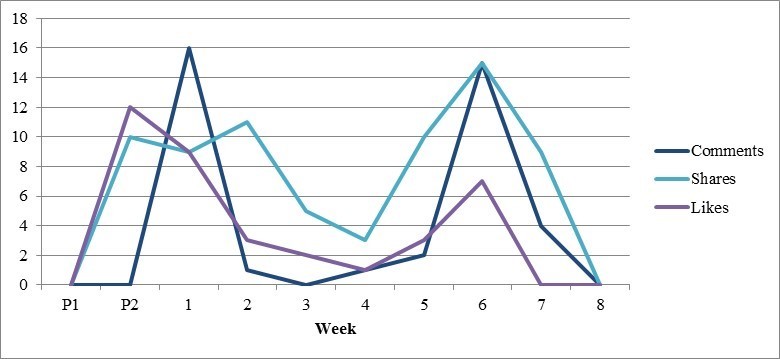
Weekly engagement with Facebook posts (phase II).

Over the course of 10 weeks, the 52 posts reached 5866 users (Table 3). Of these, 22 posts posed discussion questions to parents regarding the importance of certain outcomes related to ARI, 20 provided parent resources, and 10 were promotional. All contained images or videos, which were varied to include pictures of children, graphics, infographics, and other child-related content. We compared the three types of posts to determine whether patterns emerged in how users interacted with different content or types of engagement (Table 3). Although no strong trends surfaced, study discussion questions received the most attention, with the most comments (90%, 35/39), likes (46%, 17/37), and shares (67%, 48/72). However, on closer examination of our most successful posts (Table 4), we determined certain parent resources proved popular, with one post ranking second among the most liked posts by parents. Every Monday during the 8-week study period, we published a study discussion question that Modern Mama shared, increasing our reach for those posts. Two posts ranked among the five most successful in each category, each having a comparatively high reach and engagement through comments, likes, and shares. This is likely due to the fact that one was picked up and shared by the local children’s hospital foundation, which has an established and engaged follower base, and the other promoted local research and was shared by users, who were aware of this study and associated parent resource. We did not observe changes in engagement over time, with individual posts instead seeming to stand on their own, without evidence of an increased following as the study progressed (Figures 4 and 5).

**Table 3:** Facebook post activity summary (phase II).

Period of study			Reach		Comments		Shares		Likes		Clicks		Impressions^a^	
**Promotional period**														
	Mean activity per post (median, range)		80 (55, 35-227)		0 (0, 0)		1 (1, 0-8)		1 (0, 0-9)		4 (0, 0-28)		131 (146, 64-182)	
	**Mean activity by post type**													
		SDQ^b^ (median, range)	-^c^		-		-		-		-		-	
		Parent resources (median, range)	59 (59, 56-62)		0 (0, 0)		0 (0, 0)		0 (0, 0)		0 (0, 0)		128 (128, 112-144)	
		Study promotion (median, range)	77 (49, 35-227)		0 (0, 0)		1 (0, 0-8)		2 (0, 0-9)		5 (0, 0-28)		119 (132, 64-164)	
	Total activity across all posts		800		0		10		12		40		1307	
**Study period**														
	Mean activity per post (median, range)		121 (72, 0-901)		1 (0, 0-15)		2 (1, 0-7)		1 (0, 0-4)		7 (3, 0-62)		119 (137, 10-259)	
	**Mean activity by post type**													
		SDQ (median, range)	145 (89, 33-901)		2 (0, 0-15)		2 (0, 0-7)		1 (0, 0-3)		9 (4, 0-62)		122 (141, 14-259)	
		Parent resources (median, range)	97 (53, 0-600)		0 (0, 0-2)		1 (0, 0-7)		0 (0, 0-4)		5 (2, 0-36)		118 (138, 10-219)	
		Study promotion (median, range)	85 (55, 33-198)		0 (0, 0-1)		1 (0, 0-3)		1 (0, 0-3)		3 (3, 0-6)		134 (122, 58-235)	
	Total activity across all posts		5066		39 (17, 16, 6)^d^		62		25		293		5000	
**Summary**														
	Mean activity per post (median, range)		113 (66, 0-901)		1 (0, 0-15)		1 (0, 0-8)		1 (0, 0-9)		6 (2, 0-62)		121 (139, 10-259)	
	Total activity		5866		39 (17, 16, 6)^d^		72		37		333		6307	

^a^An impression is the number of times a post is displayed to a Facebook user, with or without being clicked.

^b^SDQ: study discussion question.

^c^Hyphen indicates no posts of this type were published during the respective study period.

^d^Breakdown of study comments, participant comments, and untraceable comments.

**Table 4:** Summary of five most successful Facebook posts in each category by specific metrics (phase II).

Post timing and type	Post content	Reach (rank)	Comments^a^ (rank)	Shares (rank)	Likes (rank)
Week 6 (Monday) SDQ^b^	Welcome to another week of the OUTCH^c^ Study! Thanks for checking us out. This week, we’re wondering about how your regular routine is disrupted when your child is sick. What happens in your family that’s out of the ordinary? Tell us below!	900 (#1)	13 (#2)	5 (#5)	3 (#3)
Week 6 (Thursday) Parent resources	Coping with croup? Check out this great interactive Web page designed for parents. Learn more about croup, what you can do to help your little one, and when you need to see a doctor so you and your child can get back to your daily routines!	600 (#2)	2 (#4)	7 (#3)	4 (#2)
Week 1 (Wednesday) SDQ	Hey Parents! Have you had to take your child to the doctor or emergency department because they were having a hard time breathing? What was important to you?	352 (#3)	15 (#1)	4 (-)	3 (#3)
Promotion Week 2 (Monday) Study promotion	Starting next Monday: weekly discussion topics on acute respiratory infections. Tell us what matters most when your child is sick!	227 (#4)	0 (-)	8 (#1)	9 (#1)
Week 3 (Monday) SDQ	Welcome back parents! Here in Alberta we’re just coming off of our Family Day long weekend, but we hope you had a relaxing weekend wherever you are! This week, we’re talking about medical treatment for your kids. What was your experience when your child was sick and needed treatment to help their breathing? What went well? What could have been better? What worried you? What was reassuring? Please tell us in the comments below!	220 (#5)	0 (-)	4 (-)	2 (-)
Week 1 (Monday) SDQ	Thanks for checking out the OUTCH Study! We want to make sure that your child’s health care is based on what’s most important to you when their breathing is affected. This week, tell us what matters when your child needs to see the doctor.	198 (-)	0 (-)	3 (-)	3 (#3)
Promotion Week 2 (Thursday) Parent resources	Croup can be scary. Learn more about how to manage it at home and when it is time to see a doctor in this storybook!	167 (-)	0 (-)	2 (-)	3 (#3)
Week 5 (Monday) SDQ	Happy Monday, Moms and Dads! This week, we’re talking about costs that might come up when your kids are sick. Tell us what has come up for you in the comments below!	141 (-)	0 (-)	7 (#2)	1 (-)
Week 2 (Wednesday) SDQ	If your child has needed tests like x-ray or blood tests when they’re sick, what has mattered to you? Tell us in the comments below!	123 (-)	0 (-)	6 (#4)	0 (-)
Week 7 (Friday) SDQ	Hey Parents, has your child had any complications when they’ve been sick with a respiratory infection? Tell us about it in the comments below.	73 (-)	4 (#3)	1 (-)	0 (-)

^a^Only four posts have been ranked in this category because all others received no comments.

^b^SDQ: study discussion question.

^c^OUTCH: OUTcomes in Child Health.

#### Stakeholder Engagement

We found that 4 parents contributed to the study discussion, of which 2 commented on more than one topic across study weeks, and 2 provided one comment each. Overall, our peak periods of activity were seen during the second promotional week and the first and sixth study weeks. Initially, members of the study team shared promotional material to encourage study involvement, driving activity levels in promotional Week 2. Week 1 encompassed an ongoing dialogue with a parent who had been recruited during the promotional period, resulting in our highest number of comments from the study. Week 6 benefitted from having one post shared by an influential organization, resulting in our highest reach and dialogue with two new study participants. The remaining weeks garnered relatively low levels of engagement. Stakeholder engagement over the study period is depicted in Multimedia Appendix 4.

Of the 33 organizations that we contacted in advance of the study, 5 provided support for promoting the study within their networks. With the exception of weekly shares by Modern Mama, each group advertised the study at one point in time. Other forms of collaboration from our research partners included tweets from TREKK and ARCHE, and a guest post published on the CAPHC blog (see Multimedia Appendix 4).

## Discussion

### Principal Findings

Social media is widely considered to be a promising set of tools in health research [25-34]. We evaluated a multicomponent Web-based approach to engage parents in outcomes research that utilized a study website, Facebook page, and Twitter account to recruit participants to a survey and online discussion. Overall, this strategy was of limited effectiveness, resulting in 110 responses to the survey and recruitment of 4 parents to the discussion component.

On the basis of the limited demographic data available, we found that phases I (survey) and II (Facebook) of our study primarily reached a homogenous audience of highly educated urban-dwelling females. In part, this likely reflects that while parents of either gender are highly likely to use social media, mothers are significantly more likely to use Facebook than fathers and spend more time engaging with the platform [35]. However, the distribution of other demographic information suggests that our strategy was not successful in bridging other geographic and socioeconomic divides. As the majority of our audience was from Canada, we compared demographic data collected from our survey to recent data from Statistics Canada, highlighting this discrepancy. In terms of family structure, 89.1% (98/110) of our sample was married or in a common-law relationship, which is similar to national data (79.9% of children aged 14 years and under lived with married or common-law parents in 2011) [36]. Yet, while 42.7% (47/110) of our sample had completed a postgraduate degree, 9.4% of Canadians aged between 25 and 64 years old had done the same [37]. Additionally, we defined a rural community as having a population of less than 10,000 people, a classification which encompassed 6.4% (7/110) of our respondents. Statistics Canada uses a more restrictive definition of rural, with a population of less than 1000 people, and in 2006, 19.8% of the Canadian population lived in rural communities, and another 12.2% lived in small population centers (1000-29,999 population) [38]. In these respects, it does not appear that we engaged with a representative population. It may be argued that it is unnecessary or unrealistic to be representative of the general population because disparities remain in social media usage across groups. Among all American adults in 2015, 54% with a high school diploma or less used social media, compared with 70% with some college education and 76% with college or graduate degrees. Similarly, 58% of rural residents used social media, compared with 68% of suburban residents and 64% of urban residents [39]. However, pediatric ARIs are widespread across all demographics; therefore, we were unable to capture data on a representative sample of our target audience.

We observed a substantial gap between the number of visitors to the study and survey websites and those that proceeded to open and complete the survey. This may have been partly due to the design of the websites. Potential improvements to encourage increased participation may include optimizing the landing page to make it more appealing, having a prominent call to action, and reducing the number of clicks required to access the survey [40]. We had intentionally designed our online strategy to include multiple platforms that we could tailor according to their different functionalities. We wanted to maintain a study website that could provide detailed information about the study, as well as act as a resource for parents seeking information on ARI. However, landing on the study website necessitated an extra step for visitors wanting to access the survey, and this may have resulted in drop-offs due to the indirect pathway to the final website of interest [41]. Similarly, to meet the requirements of the approving ethics board, the landing page of the survey website required visitors to scroll through an information sheet before opening the survey, which may have turned some users away [42]. Although we had intended to use the best features of different online tools to emphasize different aspects of the study, we may have had more success with a consolidated strategy that could ease the demand on users.

Other potential contributors to the low response rate may have included initial uncertainty about eligibility, or being drawn in by the mention of a raffle but losing interest when asked to complete the survey. The length of the survey may also have posed an issue for some people [43]. Participants took a median time of 11.3 min (2 to 308.8 min) to complete the survey, which may have presented too onerous a task for some. Shortening the survey may have subsequently increased response rates [44,45]. Research has indicated that the longer a survey, the greater the perceived cost of participation and thus the lesser the likelihood that patients will be willing to participate [46]. As participants move through a survey, their responses also tend to diminish in quality such that the longer a survey, the lower the quality of responses as participants begin to fatigue [46]. Brief surveys (ie, one or two questions) can oftentimes be just as valid as longer ones, thus providing high quality data while also enticing more individuals to participate. In future patient engagement efforts, particular attention to survey length should be paid during the planning phase.

We initially used two different social media platforms in an effort to engage with parents: Facebook and Twitter. We put in equivalent efforts in promoting the study through both platforms, but the clear preference among this population was for Facebook. This is reflective of general social media usage trends, in which 71% of American online adults were on Facebook as of September 2014 and 23% used Twitter [47]. For the second phase, we drew from this experience and limited our efforts to engaging participants via Facebook. Despite a more focused strategy, it remained difficult to obtain buy-in, both at the levels of the target audience and of the key influencers we wished to involve, and we had little engagement with the account. Daily views and clicks were minimal, and were not proportional to the time and resources required to maintain a presence. The challenges we encountered in participant recruitment may, in part, be reflected by parents’ perceived risk of participation. Minimally invasive research posing little individual risk is more appealing to potential study participants [48,49]. In light of this, obtaining buy-in using social media for research study participation may be difficult for certain health topics given the lack of confidentiality or anonymity associated with the interface. Using social media as a tool for parent engagement on scientific evidence or findings may prove less challenging, and would be an interesting topic for future research.

Recognizing the challenges associated with approaching members of the general population solely as researchers [50,51], we sought to increase credibility by partnering with health care organizations and parenting communities that were already known and trusted by our target audience; however, building the necessary connections and relationships requires a significant investment of time, challenging feasibility within the budgetary and timeline constraints of grant funding. A further consideration is that many organizations will offer support for a fee, and therefore must be taken into account when budgeting for a project. Although, in principle, the possibility of messages spreading widely and organically via social media is appealing, without an established and well-connected social media presence, the reality is likely that far more often, a much more deliberate, and resource-intensive strategy is required. In future research, leveraging existing organizations and communities with access to the parent population early, consistently, and as frequently as budgeting permits throughout the duration of the study would likely improve buy-in and facilitate parent engagement and participation. Contacting organizational partners before the study start date, sending regular, brief reminders throughout the study period, and providing some degree of mutual benefit (eg, study progress updates, providing a summary of study results) to encourage their support may also prove beneficial.

During the first phase, we received far more traffic to the survey website from the paid advertising campaign than our organic strategy, although significantly fewer surveys were completed. Although we do not have data describing all visitors to the survey website, an interesting question that arises is when users determined whether or not they were eligible for the study. A potential explanation is that the Web traffic referred to the site was not actually representative of the target audience, in which case a more tailored recruitment strategy could be necessary. The majority of respondents indicated that the source of referral to the study was either through Facebook or a friend. Although we did not specifically target existing contacts, we noticed that many of the survey respondents belonged to the professional and social networks of the study team, suggesting not only that personal connections are an important facilitator, but also that our online reach was unable to spread much beyond our existing network. To maximize the utility of social media, it will be important to break this pattern. Of completed surveys, most were returned in the early days of the study period [52-54]. This is common to closed surveys with a set sampling frame; however, in theory, we could have had ongoing recruitment. This is likely reflective of our response pattern, in which many respondents had an association with the study team, and we were unable to expand our reach much beyond close contacts.

### Strengths and Limitations

**Figure 6:**
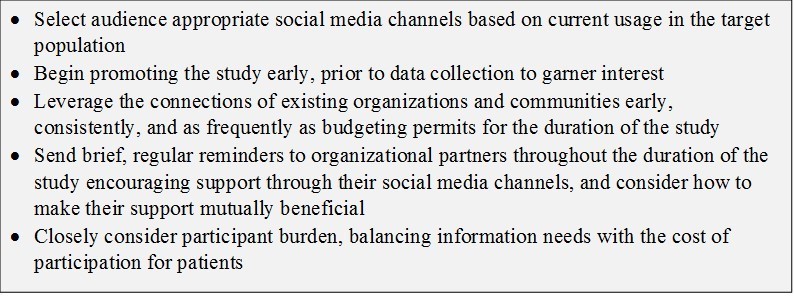
Lessons learned.

Patient-centered outcomes research is an emerging field, and the best methodologies for engaging stakeholders, including parents, have yet to be identified. Our study evaluated social media as a novel approach to engage parents in the research process, using a tailored and systematically measured strategy. Although our success in engaging participants was limited, an advantage of this project is that it highlights a number of the practical challenges associated with the use of social media for health research, providing guidance for future work in the area; specifically, the need to evaluate and compare purposive social media-based patient and family engagement strategies. Despite the fact that social media use is prevalent among our intended study population, there are nuances involved in navigating the dynamics and relationships that exist within these platforms that can make research challenging. Much of the existing evidence on the use of social media within health research has been based on ad hoc strategies and the assumption that the widespread use of social media will readily lend itself to research purposes; however, this does not capture the extent of the complexity. Within the field of patient-centered outcomes research, where stakeholder engagement is the driving component, identifying how patients and families perceive the use of social media in research, how they prefer to be involved, and whether different tools or approaches are more effective for different questions will be critical. The lessons learned from this study (Figure 6) will be of benefit to researchers seeking to engage patients in the identification of outcomes relevant to themselves and their families, as little guidance on using social media for this purpose currently exists.

The nature of social media allows for a transience and degree of control over privacy settings among users that does not permit comprehensive information to be collected on the demographic composition of the audience. Additionally, independent analytics programs were used to track metrics across each of our different online components, resulting in inconsistent data being collected from each source. Therefore, the information about our users and their activity was incomplete. A caveat to note, though, is that we used the free versions of each of these programs. Although upgrading would increase costs, it may permit a wider range of features that would allow a greater degree of harmony between data collected from different sources.

Our study was focused on parents of children with ARIs. This is a broad category, encompassing a range of conditions; therefore, we did not have a specific, well-defined end user or special interest group. Additionally, we concentrated on acute conditions. Our strategy may have had better success within a more defined condition or disease, or within a chronic condition, as it may have been more feasible to access a more readily available and already engaged network. However, respiratory conditions are common in children and patient-centered outcomes in this area are understudied, making evaluations important contributions to the evidence base.

Future research will be valuable in elucidating what makes a social media strategy effective in engaging stakeholders. In our study, we created and utilized new social media channels with no prior following. Using established social media channels to recruit and engage participants may have yielded different results (eg, increased engagement) and would be a worthwhile avenue for further exploration. The enthusiasm surrounding the potential uses of social media platforms can overshadow real constraints, including resources required, challenges in creating a network among the target audience, and navigating new territory for ethical approval. Many of the conventional ethics requirements are difficult to translate to research using social media given the vast potential reach. We did not have a defined sample that we were trying to access; therefore, issues related to sample size and participant demographics were not easily delineated upfront. Additionally, we were asked by the ethics board to provide Facebook and Twitter posts in advance, which runs counter to the evolving and interactive nature of social media platforms. Gaining exposure and continuing to evaluate aspects of social media use will help to inform the development of efficient and exciting strategies to connect with patients, their families, and the public.

### Conclusions

Our social media engagement strategy resulted in a sample of survey respondents and discussion forum participants that were highly educated, urban dwelling, and female. The source of referral for most survey respondents was through Facebook or a friend, suggesting the importance of personal connections in the dissemination and uptake of information; however, user engagement with the Facebook-based discussion threads was inconsistent and difficult to initiate. Interaction with our online presence was limited relative to the resources required to deliver the approach. The use of social media as a component of a patient engagement strategy for outcomes research is associated with several challenges, and requires thoughtful design and implementation, dedicated resources, and methods in place to ensure representativeness. Future work to evaluate effective components of social media strategies, as well as patient and family preferences for engagement using this modality, will advance the science of patient-centered outcomes research.

## Appendix

Multimedia Appendix 1 A static snapshot of the study website.

Multimedia Appendix 2 A static snapshot of the study Facebook page.

Multimedia Appendix 3 A static snapshot of the study Twitter page.

Multimedia Appendix 4 A network diagram of stakeholder engagement.

## Abbreviations

ACCFCR: Alberta Centre for Child, Family & Community Research
ARCHE: Alberta Research Centre for Health Evidence
ARI: acute respiratory infection
CAPHC: Canadian Association of Paediatric Health Centres
CCNet: Cochrane Consumer Network
IQR: interquartile range
KT Canada: Knowledge Translation Canada
OUTCH: OUTcomes in Child Health
TREKK: TRanslating Emergency Knowledge for Kids
