# Supplementary figures and images for: Outcomes in Child Health: Exploring the Use of Social Media to Engage Parents in Patient-Centered Outcomes Research
Source: J Med Internet Res. 2017 Mar 16;19(3):e78. doi: 10.2196/jmir.6655 (PMC5374273; doi:10.2196/jmir.6655)

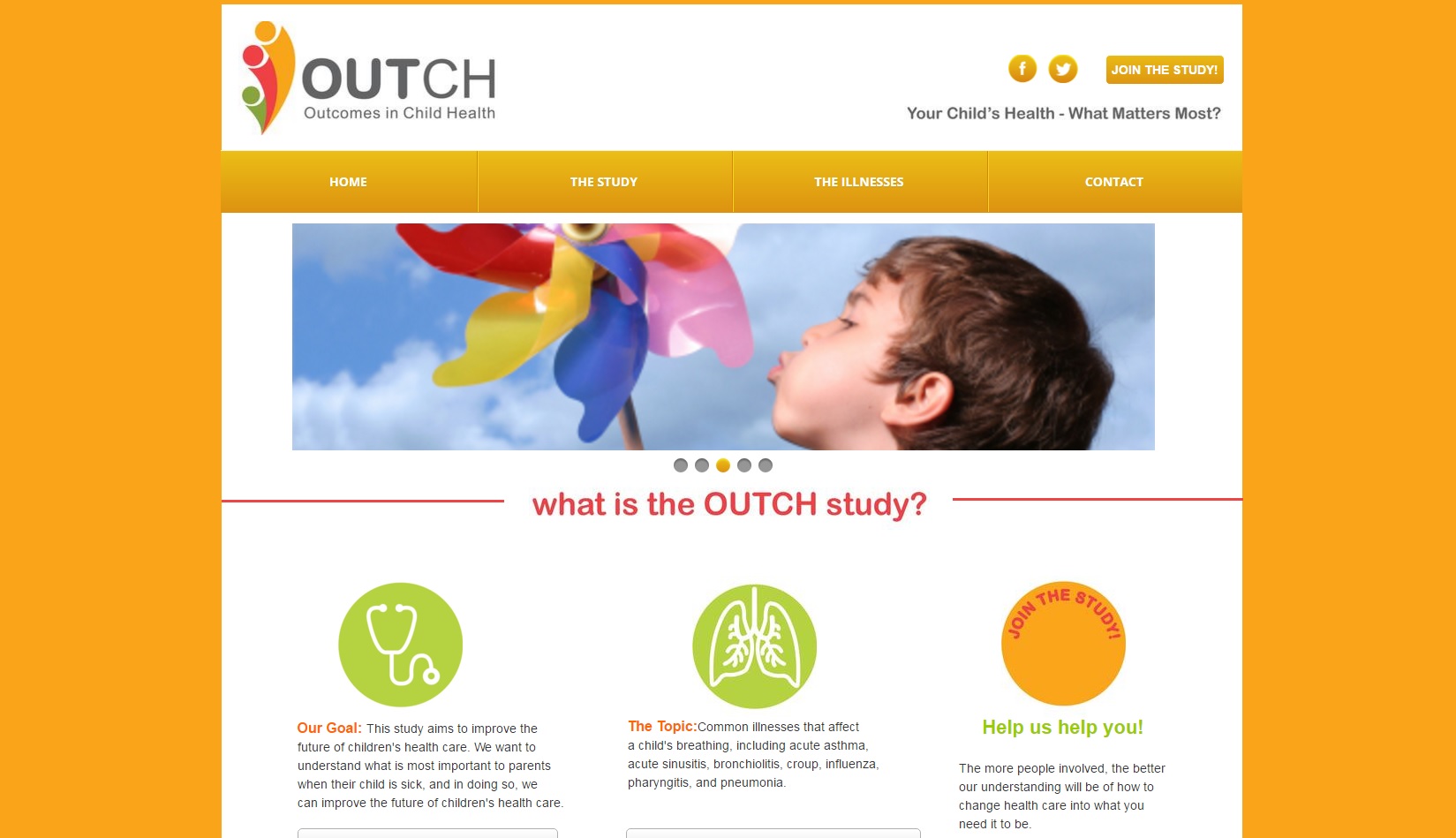

Supplement: Multimedia Appendix 1 [file jmir_v19i3e78_app1.jpg]

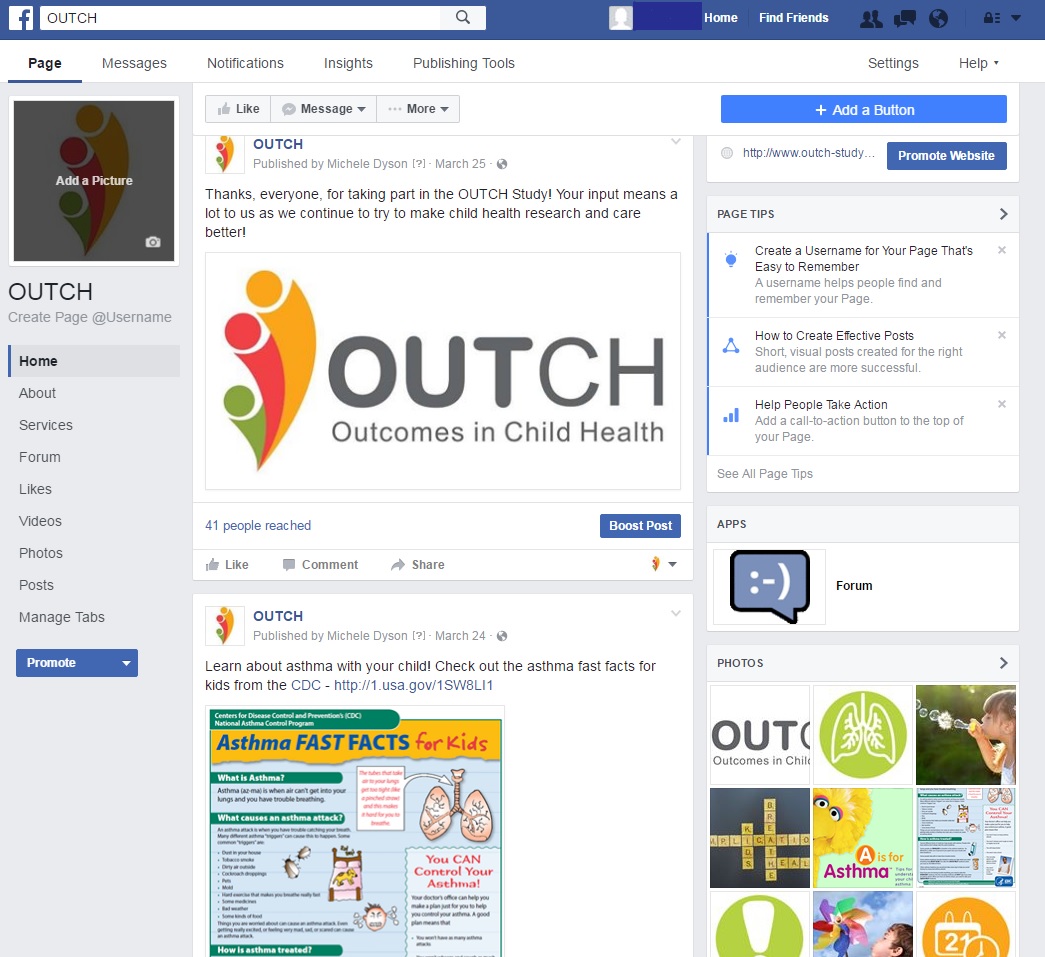

Supplement: Multimedia Appendix 2 [file jmir_v19i3e78_app2.jpg]

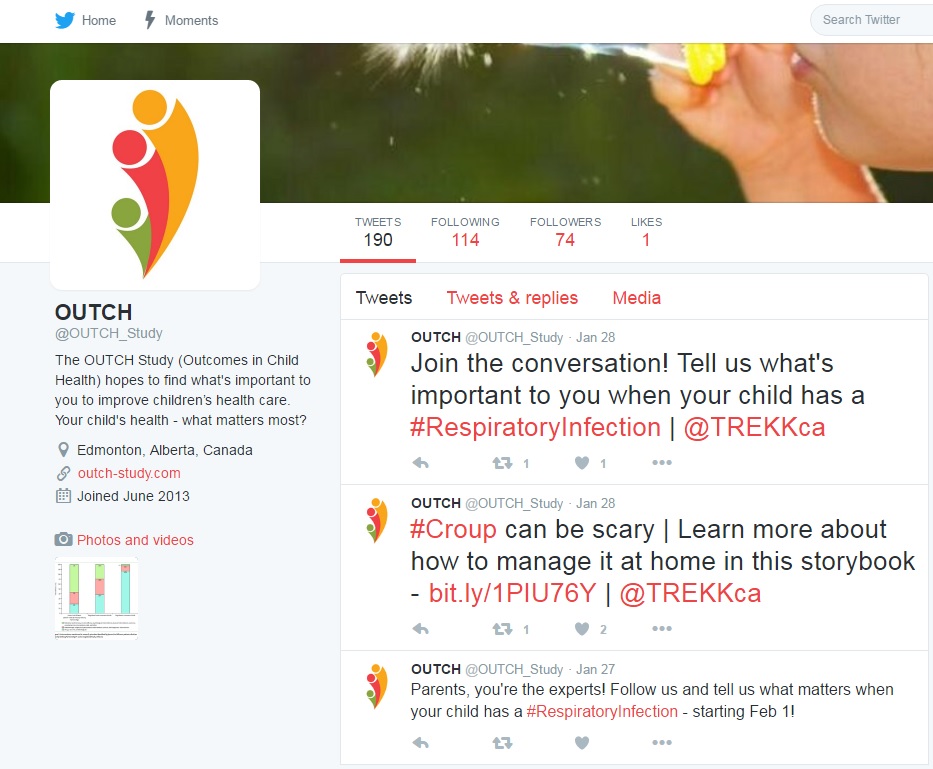

Supplement: Multimedia Appendix 3 [file jmir_v19i3e78_app3.jpg]

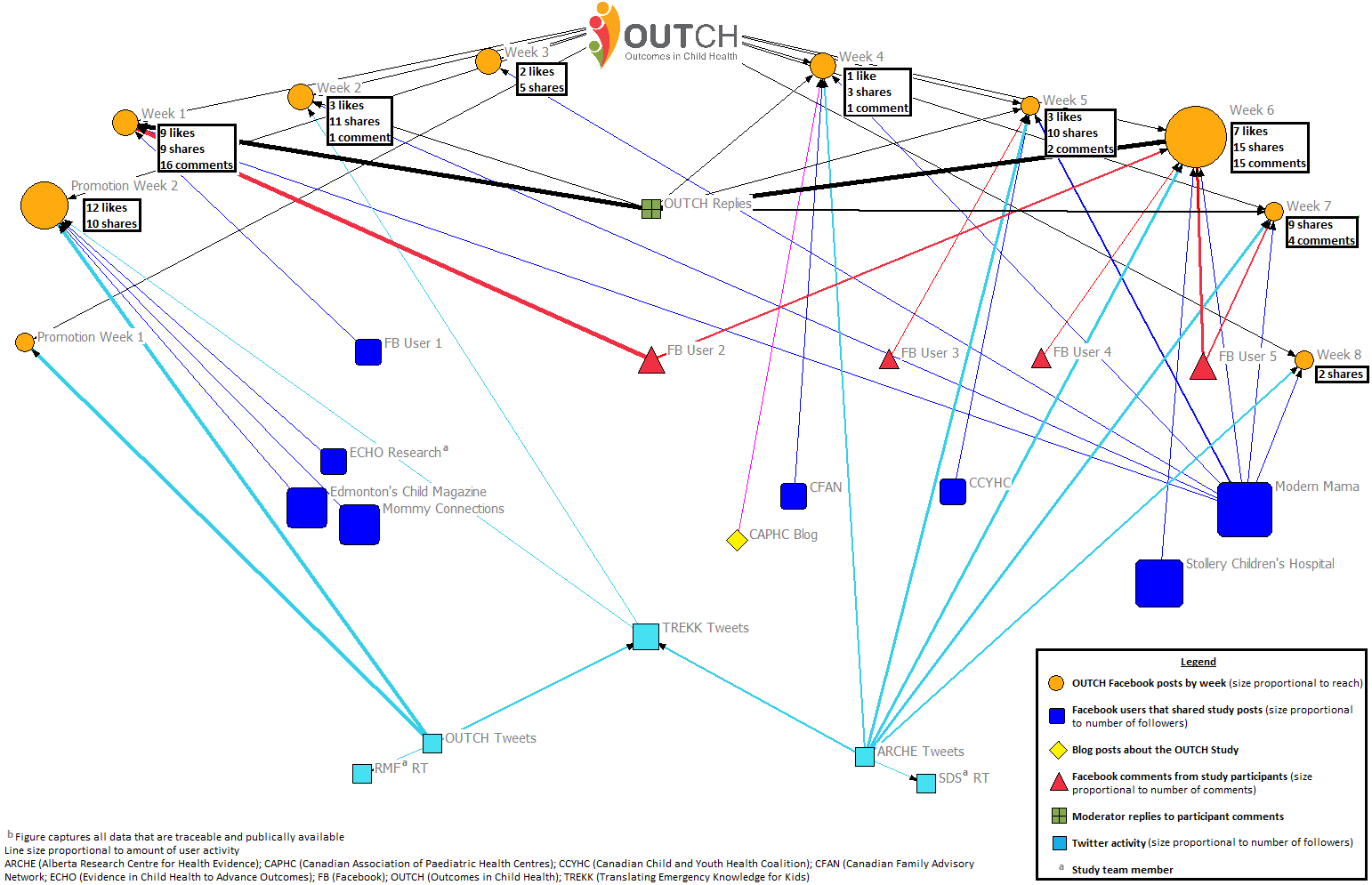

Supplement: Multimedia Appendix 4 [file jmir_v19i3e78_app4.png]
